# Pembrolizumab‐induced sarcoid granulomatous panniculitis and bullous pemphigoid in a single patient

**DOI:** 10.1002/ccr3.2090

**Published:** 2019-03-11

**Authors:** Anthony D. Honigman, Francis Lai, Joshua Elakis, Owen Prall, Michelle Goh, Christopher McCormack

**Affiliations:** ^1^ Monash Health Melbourne Victoria Australia; ^2^ Department of Dermatology Peter MacCallum Cancer Centre Melbourne Victoria Australia; ^3^ Department of Pathology Peter MacCallum Cancer Centre Melbourne Victoria Australia

**Keywords:** granulomatous panniculitis, immunotherapy, PD‐1 inhibitor, Pembrolizumab

## Abstract

Pembrolizumab is an immune checkpoint inhibitor with antitumor activity in other organ malignancies. We present this case —demonstrating multiple inflammatory adverse events associated with Pembrolizumab (in a single patient), in order to increase awareness and facilitate earlier identification of the wide‐ranging cutaneous side effects associated with immunotherapy.

Pembrolizumab is a humanized monoclonal antibody IgG4 programmed cell death‐1 (PD‐1) antagonist, immune checkpoint inhibitor approved for treatment of metastatic melanoma, with antitumor activity in other solid organ malignancies.^1^ We report a case of sarcoidal granulomatous panniculitis and bullous pemphigoid associated with Pembrolizumab in a single patient.

A 56‐year‐old woman was treated with Pembrolizumab in mismatch repair‐deficient (absent PMS2) metastatic endometrial adenocarcinoma. Four months into treatment she presented with red‐brown fixed, indurated, and tender papules and plaques on the lower legs (Figure 1), the largest plaque measuring 2 cm. Histopathology from both lower legs showed multiple small well‐formed, non‐necrotizing sarcoidal granulomata in the dermis and subcutis (Figure 2) with no foreign material or vasculitis. Periodic acid‐Schiff, Ziehl‐Neelson, Wade‐Fite, and Gram stains were negative for organisms, and *Mycobacterium tuberculosis* PCR was negative. Serum interferon‐gamma release assay for *Mycobacterium tuberculosis* (QuantiFERON‐TB Gold®) was indeterminate. Connective tissue disease screen was unremarkable. Angiotensin‐converting enzyme was elevated at 83 (12‐40 U/L) and chest computed tomography showed bilateral small subpleural pulmonary nodules and mediastinal and hilar lymphadenopathy, presenting two months after starting Pembrolizumab, stabilizing from four months. Absence of progression on serial chest imaging in correlation with granulomata of the skin, pulmonary nodules, and lymphadenopathy were thought consistent with a granulomatous process rather than metastatic disease. Skin lesions improved with topical Betamethasone dipropionate 0.05% ointment. Fourteen months into Pembrolizumab treatment, a second skin eruption with widespread pruritus and excoriated papules on limbs and torso (Figure 3) presented and biopsy confirmed bullous pemphigoid with subepidermal vesicles and eosinophils. Immunofluorescence demonstrated IgG and C3c at the dermo‐epidermal junction. Oral prednisolone induced remission, enabling continuation of Pembrolizumab.

**Figure 1:**
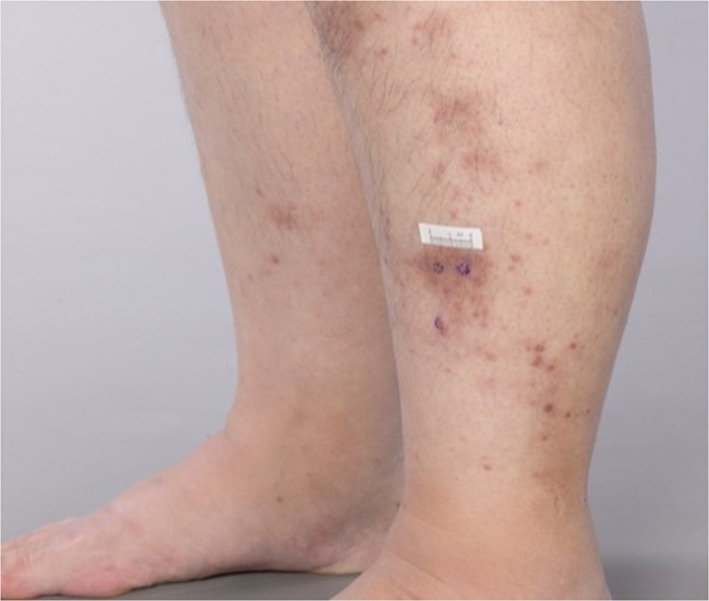
Red/brown fixed, indurated, and tender papules and plaques

**Figure 2:**
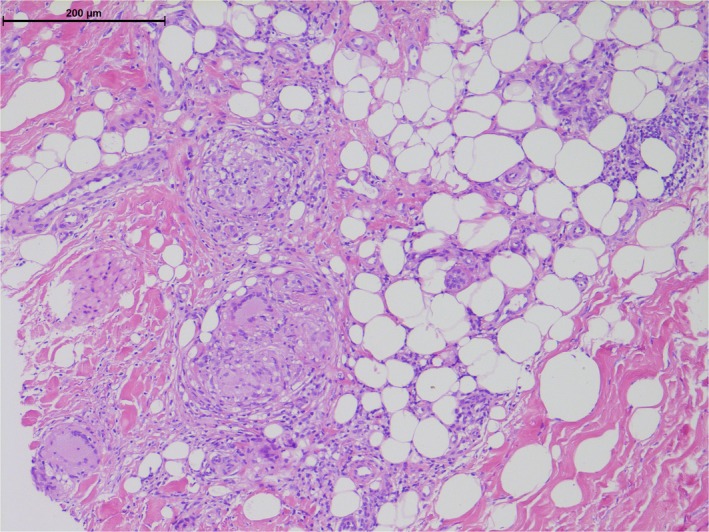
H&E stain at magnification of punch biopsy showing well‐formed, non‐necrotizing granulomas and histiocytes

**Figure 3:**
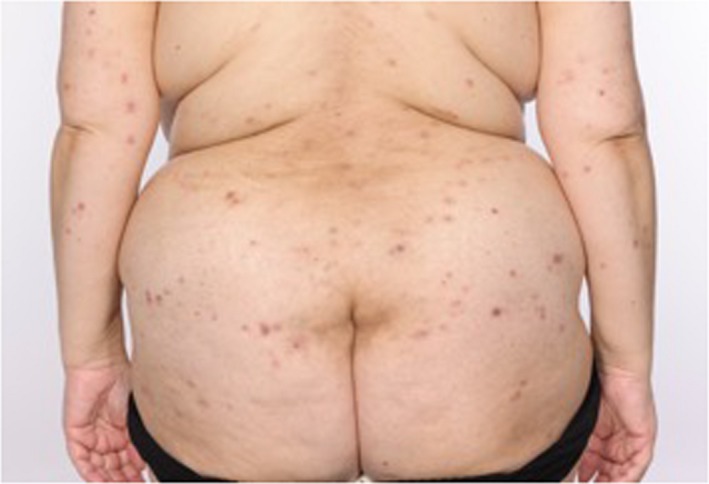
Excoriated papules on torso

PD‐1 inhibitors are a mainstay of metastatic melanoma treatment due to its efficacy,^1^ yet are implicated in multi‐system autoimmune inflammatory adverse events. They cause a release from normal immune inhibition, analogous to “releasing the brake” on immune tolerance.^2^ Adverse dermatological events such as lichen planus, lichenoid drug eruptions, dermatitis, bullous pemphigoid, acute localized exanthematous pustulosis, and Stevens‐Johnson syndrome/ Toxic epidermal necrolysis have been reported.^1^, ^3^ Some events have even been suggested as positive prognostic factors, with improvements in melanoma patient survival with Pembrolizumab.^1^

Granulomatous reactions in the form of extensive panniculitis and granulomatous inflammation reactivation affecting the lungs in metastatic melanoma patients undergoing Pembrolizumab therapy have been described.^4^, ^5^ Cases of bullous pemphigoid associated with Pembrolizumab have also been reported, many with prior treatment with Ipilimumab.^5^ In our case, we describe an individual presenting with granulomatous panniculitis as well as bullous pemphigoid associated with pembrolizumab therapy. Our report appears to be the only recorded case showing multiple cutaneous immune‐related adverse events in the same patient, expanding the clinical spectrum of cutaneous manifestations of Pembrolizumab therapy to include possibility of granulomatous panniculitis and polymorphic cutaneous autoimmune conditions in a single patient. As the use of PD‐1 inhibitors grows, clinicians must be cognizant of potential for associated immune‐mediated cutaneous adverse effects. Here we aim to increase awareness of atypical presentations of Pembrolizumab therapy to facilitate earlier identification of the wide‐ranging cutaneous side effects associated with immunotherapy.

## CONFLICT OF INTEREST

No author has any conflicts of interest or relevant financial activities to disclose.

## AUTHOR CONTRIBUTION

AH: first author and is a corresponding author. FL: involved in clinical care of patient and assisted in drafting the manuscript. JE: assisted in drafting the manuscript. OP: consultant pathologist involved in clinical care of patient and  assisted in drafting the manuscript. MG and CM: consultants involved in clinical care of patient and assisted in drafting the manuscript.
